# Developing organisational capacity building for person-centred dementia care in nursing homes: a multiphase co-design study protocol

**DOI:** 10.1136/bmjopen-2026-123820

**Published:** 2026-08-12

**Authors:** Tina Quasdorf, Sven Brenner, Iris Kramer, Kornelia Kotkowski, Johanna Gutenberg

**Affiliations:** 1 School of Health Sciences, Institute of Nursing, Zurich University of Applied Sciences, Winterthur, Switzerland; 2 Senior Health Centres of the City of Zurich, Zurich, Switzerland

**Keywords:** Dementia, Nursing Homes, Implementation Science, Person-Centered Care, Organisational development

## Abstract

**Abstract:**

**Introduction:**

Person-centred dementia care (PCdC) is widely recognised as a core principle of high-quality dementia care and is embedded in national and international dementia care guidelines. However, translating PCdC into routine practice in nursing homes (NHs) continues to pose a significant challenge. This reflects both the theoretical complexity of PCdC and the fact that its delivery relies less on discrete care techniques and more on an underlying organisational culture and shared approach to care. Implementing PCdC therefore requires an organisational perspective that acknowledges the interplay between staff, leadership and organisational conditions. Although several approaches exist to strengthen organisational capacity for PCdC, NHs often lack guidance on how to identify and apply approaches that fit their specific context. This study aims to develop a toolbox that supports NHs in systematically building the organisational capacity required to implement and sustain PCdC in their specific context.

**Methods and analysis:**

The toolbox will be developed through three consecutive studies:

Study 1: An exploratory qualitative study of the perspectives of managers and staff in NHs on organisational capacity building (OCB) for implementing and sustaining PCdC.

Study 2: A co-design study involving workshops to develop a first version of the OCB toolbox, informed by insights from a preliminary scoping review (conducted separately and published as a protocol on Open Science Framework) and Study 1.

Study 3: A qualitative consensus-building study using a nominal group technique with stakeholders and PCdC experts to refine and finalise the OCB toolbox (validation phase).

The toolbox development is conceptualised as the development of a complex intervention and will therefore be informed by O’Cathain *et al*’s guidance for developing complex health interventions. While co-design principles guide the overall development process, Study 2 represents the dedicated co-design phase involving collaborative workshops, ensuring that the resulting OCB toolbox integrates research evidence with practice-based insights.

**Ethics and dissemination:**

The Cantonal Ethics Committee Zurich confirmed that the study is exempt from formal ethical approval under the Swiss Human Research Act (Req-2025-00812). Findings will be disseminated through peer-reviewed publications, conference presentations and practice-oriented outputs, including a toolbox for NHs.

**STRENGTHS AND LIMITATIONS OF THIS STUDY**

- This study uses a systematic and theory-informed qualitative approach to develop a complex intervention, supporting conceptual clarity and methodological rigour.
- The sequential substudies allow findings to be carried forward iteratively, so that each phase informs the design and content of the next.
- Co-design principles guide the development process, ensuring active involvement of nursing home managers, staff and dementia experts, which enhances practical relevance, acceptability and contextual fit.
- The co-design process requires time and staff availability, which may limit participation and the range of perspectives represented.
- As with any complex intervention, the toolbox will require contextual adaptation when applied in other healthcare systems or cultural settings.

## Introduction

 Globally, dementia or dementia-related symptoms are common among nursing home (NH) residents, with a meta-analysis of 19 studies estimating a prevalence of 53%.^1^ In Switzerland, a 2014 report^2^ estimated that about 65% of NH residents had diagnosed or suspected dementia, and this proportion is expected to increase further given demographic trends and rising life expectancy.

Dementia is a clinical syndrome characterised by progressive deterioration in cognitive function. It results from various diseases and injuries affecting the brain, with Alzheimer’s disease accounting for 50–75% of cases.^3^ Dementia primarily affects older adults and leads to significant disability, dependency and mortality, with profound physical, psychological, social and economic impacts on individuals, families and health systems. Despite recent advances in disease-modifying treatments, particularly for Alzheimer-type dementia,^4–6^ dementia remains a progressive condition without widely available curative therapies. Current pharmacological options may slow disease progression or alleviate symptoms in some cases, but do not remove the need for comprehensive long-term care.^7 8^ The condition is associated with a complex constellation of cognitive, behavioural, psychological and functional symptoms,^3^ underscoring the importance of high-quality care focused on maintaining and improving quality of life as the primary goal of dementia care.^9–11^ Accordingly, dementia care focuses less on biomedical treatment and more on non-pharmacological approaches that support the person with dementia and address their complex needs.^12^

This shift has led to the widespread adoption of person-centred dementia care (PCdC) as a guiding approach in high-quality dementia care.

### Person-centred dementia care as the standard of care

PCdC is internationally established as the contemporary standard for high-quality dementia care.^13–19^ Rooted in Rogers’^20^ client-centred approach and adapted to dementia care by Kitwood,^21–23^ PCdC emphasises the maintenance of personhood through recognition, respect and supportive relationships.^24^ Brooker’s VIPS framework operationalises PCdC into four core dimensions: (1) valuing people with dementia and their carers, (2) individualised care, (3) understanding the perspective of the person living with dementia and (4) creating a supportive social environment.^25–27^

### Implementing PCdC in routine practice

Although person-centred approaches have been applied to dementia care since the 1990s, implementing PCdC in everyday practice remains challenging,^28 29^ particularly because implementation depends on organisational context rather than individual care techniques alone. PCdC requires a shift from task-oriented, efficiency-driven routines toward a lifeworld-oriented care approach that prioritises personhood, relationship building and meaningful engagement.^17 30^ Yet, in NHs, structural barriers such as staffing shortages, time pressure, high workloads and fragmented communication and teamwork hinder this shift. Further, insufficient staff training and knowledge, staff and management attitudes and limited organisational flexibility also hinder the implementation of PCdC.^28 31–36^ Taken together, these challenges indicate that implementing and sustaining PCdC requires a whole-systems approach. Such an approach encompasses the organisational context, including care culture, leadership, communication, decision-making processes, the physical environment and organisational flexibility.^30 37–41^ Accordingly, establishing and sustaining a person-centred care is best understood as an ongoing organisational process that requires systematic development and organisational capacity building (OCB).^30 41 42^

### Organisational capacity building

Following Cox *et al*,^43^ organisational capacity can be understood as a multidimensional construct shaped by organisational culture and communication, and expressed through six interrelated dimensions: leadership, strategy, structure and governance, skills, human capital and accountability. Building organisational capacity for PCdC therefore requires interventions that address these dimensions in ways tailored to the specific organisational context.

Despite the central role of organisational capacity in enabling and sustaining PCdC, evidence on how NHs can practically build this capacity remains fragmented. Existing reviews identify promising elements, such as staff training, cultural change, environmental modifications and organisational restructuring, but they do not focus specifically on OCB.^44–48^ Moreover, many interventions emphasise education while neglecting other dimensions of organisational capacity as described by Cox *et al*,^43^ and they rarely account for contextual factors known to influence PCdC implementation.^28 30 33–36^

Overall, practical, multidimensional and whole-system strategies for building organisational capacity to implement and sustain PCdC in NHs remain underdeveloped, particularly approaches that address key barriers and facilitators and can be adapted to diverse local contexts. The overall aim of this study is therefore to develop a toolbox that supports NHs in building organisational capacity to implement and sustain PCdC, providing practical strategies to address barriers and leverage facilitators, and enabling their context-specific adaptation and use.

## Methods and analysis

This study follows a multiphase qualitative design embedded within a co-design approach to develop a complex intervention, namely an OCB toolbox.

Based on the Medical Research Council (MRC) framework for developing and evaluating complex interventions,^49^ which comprises multiple phases, this study focuses on phase 1: *identification* and *development*. During this phase, the OCB toolbox will be developed.

As the project includes the systematic development of a complex, multicomponent intervention, the reporting of this study protocol is informed by the Guidance for reporting intervention Development studies in health research (GUIDED) recommendations^50^ (online supplemental file 1).

After an initial scoping review, which is reported elsewhere,^51^ three consecutive substudies will be conducted:

1. Qualitative study: explore NH managers and staff’s perspectives on OCB for implementing and sustaining PCdC.
2. Co-design approach: develop an initial prototype of the OCB toolbox, informed by insights from the scoping review and substudy 1.
3. Expert validation: refine and finalise the OCB toolbox using a nominal group technique with stakeholders and PCdC experts.

The scoping review and the three empirical substudies are sequential and build on one another, allowing for iterative refinement of the developing intervention. Methodological adaptations may therefore occur throughout the development process in response to emerging evidence and stakeholder feedback.

To ensure transparency and traceability, we will maintain an intervention development log documenting key decisions, iterations, and how evidence and stakeholder input informed modifications to the OCB toolbox.

The overall study runs from August 2025 to January 2029. The first phase is a scoping review, which is reported elsewhere.^51^ This protocol covers the empirical sub-studies:

- Substudy 1 (August 2026 to September 2027): recruitment is ongoing at the time of manuscript submission, with completion expected by October 2026.
- Substudy 2 (August 2027 to September 2028): recruitment is planned for spring 2027, drawing on the participant pool established in substudy 1.
- Substudy 3 (August 2028 to January 2029): recruitment is planned for spring/summer 2028.

### Intervention

The proposed OCB toolbox is conceptualised as a complex intervention characterised by multiple interacting components targeting different organisational levels, requiring diverse skills and behaviours from those involved in its use and implementation, and allowing for flexibility and adaptation to local contexts.^49^ Its complexity further arises from the dynamic interactions between the intervention and the organisational environment.^52^ In line with contemporary perspectives, the toolbox is not viewed as a discrete, self-contained entity but as an intervention embedded within a complex organisational system, whose effects emerge through ongoing interactions, adaptation and co-evolution with its context.^52^ Following the recommendations of the MRC’s framework for developing and evaluating complex interventions,^49^ implementation considerations are integrated throughout all stages of intervention development, ensuring early and continuous attention to contextual factors, implementation strategies and real-world applicability.

The final format, content and structure of the toolbox will be iteratively defined through the co-design process and may be adapted in response to emerging findings.

### Methodological and theoretical frameworks

**Figure 1:**
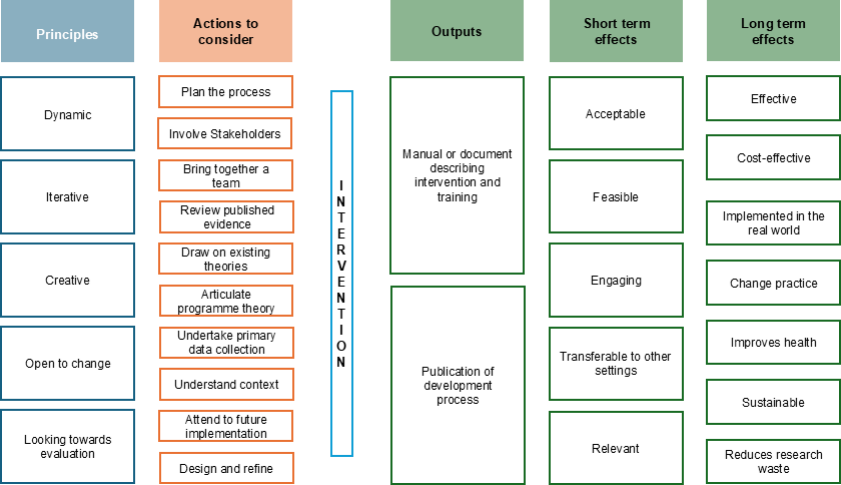
Logic model for intervention development. (Adapted from O’Cathain *et al*, p4^53^; redrawn by the authors. Author(s) 2019. Re-used under CC BY 4.0).

O’Cathain *et al*’s^53^ guidance on developing complex interventions provides the methodological framework for this study (figure 1). The guidance provides a logic model for intervention development, outlining key principles (dynamic, iterative, creative, open to change and forward-looking), which will be considered as fundamental assumptions throughout the research process of this study.

The guidance also outlines several actions to consider when developing interventions. As recommended by O’Cathain *et al*,^53^ the relevance and importance of each action outlined will be considered at the outset and continuously during intervention development. As the intervention development process progresses, the different substudies address different subsets of these ‘actions to consider’ most relevant to their respective aim and phase.

The conceptual basis for organisational capacity in this study is informed by the framework developed by Cox *et al*,^43^ which conceptualises organisational capacity as a multidimensional construct shaped by organisational culture and communication. Capacity is expressed through six interrelated dimensions: leadership, strategy, structure and governance, skills, human capital and accountability. In this study, the framework serves as the theoretical framework, providing the foundation for understanding organisational conditions relevant to implementing and sustaining PCdC, structuring data collection across the substudies, and informing the organisation and content of the toolbox components.

### Methodological approach

A multiphase qualitative co-design approach will guide all substudies to enhance the practical relevance of findings.^54 55^ Accordingly, relevant stakeholders will be engaged throughout the research process.^56^ This approach is operationalised through close collaboration with a practice partner. Two designated representatives of this practice partner will be actively involved in study design, research question specification, instrument development, data analysis and interpretation, and will serve as the interface between practice and research.

While co-design principles are applied across the entire research programme, substudy 2 represents the dedicated co-design phase. In this phase, collaborative workshops with stakeholders will be used to iteratively develop and refine the OCB toolbox, ensuring that it integrates research evidence with practice-based insights.

In the following section, we outline, separately for each substudy, the study design and research methods, thereby clarifying how each component informs and strengthens the overall research programme.

Throughout the study, trustworthiness^57^ will be enhanced. *Credibility* and *dependability* will be supported through researcher triangulation, iterative coding discussions, transparent documentation in the intervention development log and regular consultation with an external scientific advisory board comprising senior researchers with expertise in dementia care and implementation science. *Confirmability* will be strengthened through reflexive practice, with ongoing reflective discussions within the research team to consider how professional and personal backgrounds may influence data collection, analysis and interpretation.

#### Patient and public involvement

Patients and the public were not involved in the design, conduct, reporting or dissemination plans of this study.

### Substudy 1: exploration of NH managers and staff’s perspectives on OCB for implementing and sustaining PCdC

#### Study aim

The aim of this substudy is to explore the perspectives of NH managers and staff in German-speaking Switzerland on OCB to implement and sustain PCdC.

#### Research questions

1. From the perspective of NH managers and staff, what requirements are necessary to build organisational capacity to implement and sustain PCdC?
2. From the perspective of NH managers and staff, what are the organisational barriers to and facilitators for implementing and sustaining PCdC? *(implementation barriers/facilitators)*
3. Which OCB strategies and approaches do NH managers and staff consider promising for implementing and sustaining PCdC? *(implementation strategies)*

#### Study design

An exploratory qualitative study will be conducted. The study is informed by a phenomenology-oriented perspective^58 59^ focusing on how managers and staff experience and make sense of implementing and sustaining PCdC. However, rather than aiming to generate a formal phenomenological account, the study applies qualitative content analysis to systematically examine participants’ perspectives.^60^

#### Setting and participants

The study will be conducted in 10 NHs in German-speaking Switzerland, including facilities with a focus on dementia care and PCdC.

Sampling will be conducted at two levels: organisational (NHs) and individual (participants within NHs). At the organisational level, a purposive sampling strategy with a focus on maximum variation will be applied. NHs will be selected based on predefined criteria, including canton, geographical location (urban, semi-urban, rural), organisational size (operationalised by number of beds), provider type (eg, public, private, non-profit) and level of experience with PCdC. Both NHs with extensive experience and those with limited or emerging implementation of PCdC will be included to ensure a broad range of organisational contexts.

Within each participating NH, approximately 7–9 managers and 7–9 staff members will be included, covering all major service areas (nursing care, social care, therapeutic professions, physicians, food services, facility management and administration). The manager sample will include different hierarchical levels. Additionally, 14–18 dementia experts will be included from across participating NHs.

Participants will be required to have sufficient proficiency in German to participate and be aged 18 years or older. They will be purposively selected to ensure representation across professional groups, service areas, hierarchical levels, and diverse professional backgrounds and experience.

The planned sample size (approximately 10 NHs and 150–190 participants) was defined a priori. In line with qualitative research standards, its adequacy will be assessed with reference to the concept of information power,^61^ and minor adjustments may be made if indicated by emerging data needs.

#### Recruitment

Four NHs will be provided by the practice partner; the remaining homes will be recruited through established networks of the scientific and practice partners and the Scientific Advisory Board. Participants will be recruited via designated contact persons in each home.

#### Data collection and analysis

Data will be collected through semi-structured focus groups with approximately 7–9 participants each. Focus groups offer key advantages, including helping participants articulate and clarify their views, fostering synergy and spontaneity and requiring fewer resources than individual interviews.^62^ In each NH, one focus group with managers and one with staff will be conducted, complemented by two additional focus groups with dementia experts across all sites. Interview guides will be developed based on the scoping review and preliminary theoretical considerations and refined during data collection. They will explore participants’ experiences with PCdC in general, as well as organisational requirements, barriers, facilitators and strategies related to its implementation and sustainability. Each focus group will be moderated by one researcher, with a second researcher taking field notes. Sessions will last 1.5–2 hours, be digitally recorded, transcribed verbatim and pseudonymised. Data will be analysed using qualitative content analysis,^63^ supported by the software MAXQDA.^64^

### Substudy 2: development of the OCB toolbox

#### Study aim

The aim of this substudy is to develop a first version of the OCB toolbox, along with an accompanying programme theory and implementation strategy. In addition to ensuring its usefulness and relevance for practice, the toolbox is developed in line with the anticipated outcomes described in the Consolidated Framework for Implementation Research Outcomes Addendum (adoptability, implementability and sustainability), supporting the development of an intervention that is feasible and sustainable in real-world NH settings.^65^

#### Research questions

The research questions address (1) content, (2) design, and (3) implementation strategy of the OCB toolbox.

1. Which *content elements* do managers, staff members and dementia experts consider essential to ensure the OCB toolbox’s usefulness and relevance, and to support its adoptability, implementability and sustainability?
2. Which *design elements* do managers, staff members and dementia experts consider essential to ensure the OCB toolbox’s usefulness and relevance, and to support its adoptability, implementability and sustainability?
3. What do managers, staff members and dementia experts consider essential for developing a modular, context-sensitive and tailorable implementation strategy that supports the sustainable implementation and scale-up of the OCB toolbox in NHs?

#### Study design

A co-design approach^56^ will guide the development of a toolbox aligned with the needs, priorities and everyday realities of NHs. Building on the co-design involvement across all substudies, this phase represents the core co-design component of the research programme. It is operationalised through structured, collaborative workshops with stakeholders to iteratively develop and refine the toolbox. To develop the accompanying programme theory, the study will also draw on a realist approach^66 67^ to explore how contextual factors, underlying mechanisms and anticipated outcomes interact and shape the OCB toolbox’s usefulness and impact.

#### Setting and participants

The study will involve participants recruited from substudy 1, conducted in NHs in German-speaking Switzerland. Thus, the organisational context and sampling framework established in substudy 1 will be retained.

Approximately 10 managers, 10 staff members and 10 dementia experts will be included. Where feasible, 1 manager and 1 staff member will be selected from each participating NH. Participants will be required to have sufficient proficiency in German to participate and be aged 18 years or older.

#### Recruitment

Recruitment will occur among participants of substudy 1. During the consent process, individuals will be asked whether they agree to be contacted for substudy 2; those who consent will form the recruitment pool.

From this pool, participants will be purposively selected to achieve the target sample and to ensure diversity across professional roles, experience and organisational contexts.

#### Data collection and analysis

The OCB toolbox will be developed iteratively through a series of workshops. In each workshop, synthesised findings from the scoping review and substudy 1 will be presented and discussed in relation to this substudy’s research questions. Separate workshops will be conducted with managers, staff members and dementia experts. Outputs from these workshops will inform an initial draft of the toolbox, which will then be refined in a subsequent workshop involving representatives from all stakeholder groups.

Workshop notes and materials will be reviewed and thematically synthesised using MAXQDA^64^ to identify key content, design and implementation requirements. Building on this, and informed by an initial programme theory based on Cox’s^43^ model of organisational capacity, the analysis will also draw on realist principles^66^ to explore contextual conditions, underlying mechanisms and anticipated outcomes of the toolbox. These insights will guide iterative refinement of both the programme theory and the evolving OCB toolbox.

### Substudy 3: expert validation of the OCB toolbox

#### Study aim

This substudy aims to validate and refine the OCB toolbox, the accompanying programme theory and the implementation strategy in collaboration with experts from dementia care practice and other relevant stakeholder groups (eg, providers, policymakers, payers, researchers).^68^ Consistent with substudy 2, the focus will be on the toolbox’s overall usefulness and benefit and, more specifically, on its adoptability, implementability and sustainability.

#### Research questions

As in substudy 2, the research questions address (1) content, (2) design and (3) implementation strategy of the OCB toolbox. From the experts’ perspective, they examine how well the toolbox aligns with the contextual conditions and needs of NHs and what adaptations are required.

1. How well does the *content* of the OCB toolbox align with the contextual conditions and needs of NHs, and what adaptations are required to ensure its adoptability, implementability and sustainability?
2. How well does the *design* of the OCB toolbox align with the contextual conditions and needs of NHs, and what adaptations are required to ensure its adoptability, implementability and sustainability?
3. How well does the proposed implementation strategy align with the contextual conditions and needs of NHs, and what adaptations are required to ensure the sustainable implementation and scale-up of the OCB toolbox?

#### Study design

The Nominal Group Technique (NGT)^69^ will be used to validate the OCB toolbox, the programme theory and the implementation strategy with experts. This structured consensus method ensures that all participants can contribute their views and that each perspective is considered in the group’s deliberations.^70^

#### Setting and participants

The substudy will involve 6–8 dementia care practitioners from NHs and 6–8 stakeholders from the wider dementia care field.

Dementia care practitioners will be recruited from NHs not involved in earlier substudies and will represent diverse professional roles and hierarchical levels.

Stakeholders from the broader dementia care field will include policymakers, researchers and representatives of advocacy organisations, interest groups or provider networks, ensuring a diverse range of perspectives relevant to the validation of the OCB toolbox.

Participants will be required to have sufficient proficiency in German to participate and be aged 18 years or older. Participants will be purposively selected to ensure variation in professional backgrounds, experience and perspectives.

#### Recruitment

Recruitment will be conducted through the networks of ZHAW, the practice partner, and the Scientific Advisory Board. Potential participants will be identified and contacted via email or through existing professional connections.

#### Data collection and analysis

Two NGT sessions will be conducted: one with experts from dementia care practice and one with stakeholders from the broader dementia care field. Each session will last approximately 4 hours and will be facilitated by members of the research team.

Participants will receive the draft OCB toolbox, programme theory and implementation strategy prior to the NGT session and will be invited to review these materials in preparation for the workshop.

Each session will follow the four core stages of the NGT^70^:

- Silent generation: participants individually reflect on one or two guiding questions.
- Round robin: participants present ideas sequentially to the group.
- Clarification: ideas are discussed, grouped, refined or merged with agreement from all participants.
- Voting: participants rank their preferred ideas using a structured rating sheet.

The prioritised ideas from both sessions will be documented, analysed descriptively and used to inform the further refinement of the OCB toolbox, the programme theory and the implementation strategy.

## Ethics and dissemination

### Ethics considerations

This study addresses ethical, legal and social aspects (ELSA)^71^ in accordance with the Declaration of Helsinki and the Guidelines for Safeguarding Good Scientific Practice.^72^ As the research focuses on organisational development and involves stakeholders as co-design partners and experts rather than research subjects, no foreseeable risks arise from participation. The Cantonal Ethics Committee of Zurich issued a declaration of non-responsibility (Req-2025-00812), confirming that the study does not fall under the scope of the Swiss Human Research Act. All participants will be informed about the study’s purpose, procedures and data use. Participation will be voluntary, and written informed consent will be obtained. Participants may withdraw from the study at any time without consequences.

The study follows an approved data management plan, which outlines procedures for secure data handling throughout the project. All data will be stored on secure institutional servers with access restricted to authorised project staff and processed exclusively on institution-managed devices in accordance with data protection policies. Regular backups, access controls and technical-organisational safeguards will ensure data integrity; any paper documents will be kept in locked storage. No sensitive personal or health data will be collected.

Cleaned datasets and relevant documentation (eg, transcripts, workshop materials, coding files) will be archived for 10 years; raw audio files and other data not required for further analysis will be deleted at the end of the project. A cleaned and anonymised qualitative dataset may be made available in a recognised non-commercial repository that supports Findable, Accessible, Interoperable, and Reusable (FAIR) principles, provided that participant anonymity can be fully ensured. If deposited, the dataset will be accompanied by appropriate metadata and a README file, and publications will reference the repository and DOI.

### Dissemination

The study findings will be disseminated to dementia and healthcare practice as well as to the scientific community through target group-specific formats, including publications in professional and scientific journals and presentations at national and international conferences. A practice-oriented OCB toolbox will be developed and made available to the professional public. Dissemination will also target participating and collaborating care institutions, dementia care organisations and involved practitioners, supported by the networks established during the study. No identifiable participant information will be included in any dissemination materials.

Dissemination will take place throughout the project and after study completion. A preliminary publication plan has been developed and agreed on within the research team. It will serve as a dynamic document to guide and structure dissemination activities and will be adapted as needed. Study outputs will be published in open access venues whenever possible to ensure broad accessibility.

## Supplementary material

10.1136/bmjopen-2026-123820 online supplemental file 1
